# The role of tumor-infiltrating lymphocytes in triple-negative breast cancer and the research progress of adoptive cell therapy

**DOI:** 10.3389/fimmu.2023.1194020

**Published:** 2023-05-18

**Authors:** Ruonan Li, Lili Cao

**Affiliations:** ^1^ Oncology Department, The First Affiliated Hospital of Shandong First Medical University & Shandong Provincial Qianfoshan Hospital, Jinan, China; ^2^ Shandong Provincial Key Laboratory for Rheumatic Disease and Translational Medicine and Shandong Lung Cancer Institute, Jinan, China

**Keywords:** triple-negative breast cancer (TNBC), tumor-infiltrating lymphocytes (TILs), adoptive cell therapy (ACT), breast cancer, solid tumor

## Abstract

The treatment outcome of breast cancer is closely related to estrogen receptor (ER), progesterone receptor (PR), and human epidermal growth factor receptor 2 (HER2) expression. Triple-negative breast cancer (TNBC) lacking ER, PR, and HER2 expression has limited treatment options and a poor prognosis. Tumor-infiltrating lymphocytes (TILs) play a role in promoting or resisting tumors by affecting the tumor microenvironment and are known as key regulators in breast cancer progression. However, treatments for TNBC (e.g., surgery, chemotherapy and radiotherapy) have non-satisfaction’s curative effect so far. This article reviews the role of different types of TILs in TNBC and the research progress of adoptive cell therapy, aiming to provide new therapeutic approaches for TNBC.

## 1 Introduction

Breast cancer is the most common cancer in women worldwide. The International Agency for Research on Cancer estimates that the incidence of breast cancer will increase by more than 40% and the mortality rate will increase by more than 50% by 2040 (1). Breast cancer can be characterized into four subtypes: luminal A, luminal B, human epidermal growth factor receptor 2 (HER2)-enriched, and triple-negative breast cancer (TNBC) subtypes (2). Among them, TNBC with negative immunohistochemical results for estrogen receptor (ER), progesterone receptor (PR), and HER2 in the breast cancer tissue (3) is a highly heterogenous disease with an extremely poor prognosis. TNBC accounts for 15–20% of breast cancers (4). Because of its unique phenotype, TNBC is mainly treated by cytotoxic chemotherapy.

Increasingly more studies in recent years have shown that tumor-infiltrating lymphocytes (TILs) are associated with the progression of TNBC. Among patients with metastatic breast cancer, those with high-level TILs tend to have better therapeutic outcomes (5). TILs are mainly composed of T cells, B cells, and natural killer (NK) cells and react with tumor cells and non-TILs of breast cancer patients in various ways, promoting or resisting tumors and affecting the prognosis of the patients (6). Hence, further research on the role of TILs in TNBC helps provide new approaches for the treatment of TNBC.

## 2 TIL subsets interact and coordinate to build a more rigorous anti-tumor immunity against the tumor microenvironment

**Figure 1:**
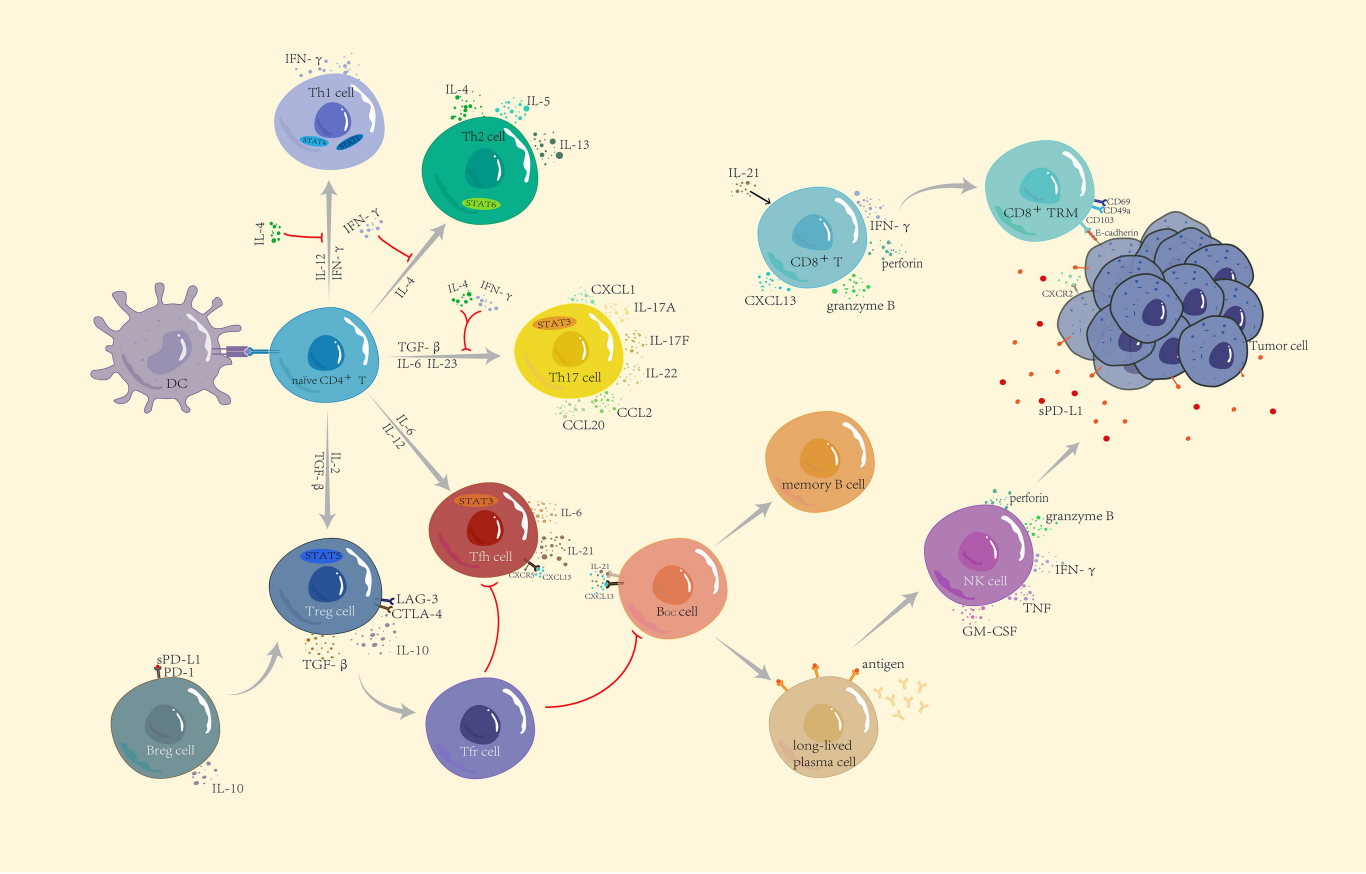
Functional subsets of CD4^+^ T cells and the role of other lymphocytes in immunity. Naïve CD4+ T cells differentiate into five subtypes: Th1, Th2, Th17, Treg, and Tfh, through the corresponding STAT pathway and cytokine induction. IFN-γ and IL-4 produced by anti-tumor Th1 cells and tumor-promoting Th2 cells not only restrict each other to maintain balance but also jointly inhibit the differentiation of Th17 cells. Th17 cells have a dual role—according to the different TMEs to secrete CCL2 and CCL20 to enhance immunosuppression or secrete CXCL1 to directly act on the surface of tumor cells to promote tumor progression. CXCL13 secreted by CD8^+^ T cells not only recruits Tfh but also participates in B cell differentiation together with IL-21. Differentiated and mature long-lived plasma cells release corresponding antibodies and upregulate NK cells to kill tumor cells. CD8^+^ T cells differentiate into tissue-resident memory CD8^+^ T cells to recognize E-cadherin on the surface of tumor cells and inhibit tumor growth. Breg and Treg cells are both tumor-promoting cells. Breg cells release IL-10 and recognize soluble PD-L1, which jointly induce Treg cell differentiation, suppress immune responses, and build a microenvironment which is conducive to tumor growth.

With the participation of the extracellular matrix, cellular components, such as tumor cells, vascular endothelial cells, pericytes, immune cells, bone marrow-derived cells, and tumor-associated fibroblasts form a relatively dynamic environment, the TME, through the release or induction of cell-signaling proteins (e.g., cytokines and chemokines). The interaction between tumor cells and the TME has a fundamental impact on cancer initiation, progression, and therapeutic efficacy (7). The rapid proliferation of tumor cells creates a hypoxic environment in the body, upregulates the expression of hypoxia-inducible factors, accelerates the recruitment of tumor cells and other related cell components to the TME, and ultimately promotes the development and metastasis of cancer cells (8). For example, hypoxia affects the release of exosomes, thereby promoting the secretion of various pro-angiogenic factors (e.g., vascular endothelial growth factor (VEGF), angiopoietin 1, and matrix metalloproteinase 9) in human umbilical vein endothelial cells (9, 10), stimulating the proliferation and migration of cancer cells and regulating tumor angiogenesis. VEGF inhibits the effect of antigen-presenting cells and effector T cells or activates immunosuppressive cells such as T regulatory cells and myeloid-derived suppressor cells and enhances the invasion effect of tumor-associated macrophages, thereby jointly suppressing the immune responses of the body (11). Nevertheless, a high TIL density in patients with TNBC is usually correlated with longer survival (12). Among the TIL subsets, T cells account for up to 75% of TILs (13). As the lymphocytes with the largest proportion in invasive breast cancer, CD8^+^ T cells are indirectly recruited by NK cells, which are only 5% of TILs, to form an important line of defense for anti-tumor immunity (14–16). B cells serve as antigen-presenting cells for T cells, eliciting localized T-cell responses in tumors (6) (**Figure 1**).

### 2.1 As a good prognostic marker, TILs provide a basis for subsequent diagnosis and treatment of TNBC

#### 2.2.1 T cells cooperate with each other to push the TME to the peak of tumor growth inhibition

T cell–mediated immunity plays a leading role in the anti-tumor process. After positive selection, T cells differentiate into CD4^+^ T and CD8^+^ T cells. Naïve CD4^+^ T cells differentiate into five subtypes: T helper (Th)1, Th2, Th17, T regulatory (Treg), and T follicular helper (Tfh) cells. Th1-secreted interferon gamma (IFN-γ) not only induces the activation of signal transducer and activator of transcription (STAT)1 and STAT4 to promote the differentiation of more Th1 cells but also recruits CD8^+^ T cells, NK cells, and other immune cells to enhance anti-tumor immunity, while Th2 cells with tumor-promoting effects mainly depend on the induction of IL-4 for differentiation (17). Importantly, IFN-γ inhibits the differentiation of Th2 cells, and interleukin (IL)-4 inhibits the differentiation of Th1 cells, thereby mutually restricting each other to maintain balance in the tumor. IFN-γ and IL-4 inhibit the development of Th17 cells (18). In the absence of these two cytokines (IFN-γ and IL-4), naïve CD4^+^ T cells are polarized into Th17 cells under the co-induction of TGF-β, IL-6, and IL-23 and subsequently secrete IL-17A, IL-17F, and IL-22 and trigger the inflammatory response in the body; moreover, Th17 cells induce the production of chemokines, such as C-C motif ligand 2 (CCL2) and CCL20 to promote macrophages and activate T cells to infiltrate the TME (19, 20). In addition, Th17, as a novel prognostic marker, was directly associated with improved overall survival of patients with non-inflammatory TNBC (21). However, a previous study showed that Th17 cells produced C-X-C motif chemokine ligand 1 (CXCL1) during breast cancer progression, which enhanced cancer cell invasion (22). As another subtype of CD4^+^ T cells, Tfh cells exist in secondary lymphoid tissues and recognize CXCL13 released by CD8^+^ T cells through regulating C-X-C chemokine receptor type 5 (CXCR5) to mediate Tfh cell recruitment. Tfh cells also secrete IL-21 to stimulate the differentiation of CD8^+^ T cells to participate in cytokine production and play cytotoxicity. As the main helper cells of tumor-infiltrating B cells, Tfh cells participate in germinal center formation and B cell differentiation, and control the humoral immune response by interacting with T follicular regulatory cells that differentiate from Treg cells. When the equilibrium shifts to the side with Tfh cells, they activate humoral immunity to actively kill tumors (23, 24).

Treg cells are a T cell subtype that promotes immune escape of tumor cells. Because of a relatively high immunogenicity, TNBC has higher Treg cell infiltration. A study by Bai et al. showed that targeting Annexin A1 downregulated CD25, C-C chemokine receptor 8 (CXCR8), and programmed cell death protein 1 (PD-1) expression and reduced the function of Treg cells, thereby enhancing anti-tumor immunity in TNBC (25). However, some scholars believe that Treg cells have a positive impact on the prognosis of patients with breast cancer. Cai et al. showed that Foxp3^+^ and Foxp3^−^ Treg cells were found in CD4^+^CD25^+^ TILs in 17 patients with TNBC (12). Foxp3^+^ circulating T cells expressed higher levels of cytotoxic T lymphocyte antigen 4, lymphocyte activation gene 3, and transforming growth factor-beta (TGF-β) after T-cell receptor (TCR) stimulation, while Foxp3^−^ circulating T cells produced high levels of IL-10, enhancing the immune effect of CD8^+^ TILs (26).

CD8^+^ T cells recognize specific antigenic peptides on the surface of tumor cells and release large amounts of IFN-γ, granzyme B, and perforin to destroy tumor cells. Surprisingly, the higher expression of CD8^+^ T cell is significantly correlated with better survival in TNBC (27). CD103 is composed of the integrin subunit αE and integrin β7. CD8^+^ T cells co-expressing CD103, CD69, and CD49a are called tissue-resident memory T cells (28). As an E-cadherin receptor, CD103 further interacts with E-cadherin to promote the activation and migration of T cells. Shields et al. showed that E-cadherin enhanced the expression of CD103 in melanoma. Under the combined actions of mature B and T lymphocytes, exogenous expression of E-cadherin delayed tumor growth, reduced tumor metastasis, and improved the survival of patients with melanoma (29). In contrast, E-cadherin deficiency promoted tumor growth and metastasis. However, the expression and anti-tumor activity of E-cadherin on CD103 in TNBC have not been verified, but some studies have preliminarily shown that CD103 and E-cadherin exist in TNBC as good prognostic markers (30, 31).

#### 2.2.2 Are B cells “friend” or “foe”?

Humoral immunity has increasingly shown a crucial role in the TME in recent years. B cells are effector cells that are mainly responsible for humoral immunity. They are activated by antigen stimulation and co-stimulatory signals. Under the action of CXCL13, the interaction between follicular dendritic and Tfh cells and B cells triggers the germinal center response, which induces the differentiation of activated B cells into memory B cells and long-lived plasma cells (32). When antigens are presented to B cells, B cells recognize and secrete the corresponding antibodies, which stimulate NK cells to naturally kill target tumor cells.

Studies have shown that TNBC is rich in highly activated B cells. By enhancing the expression of CXCL13, CXCR4, CCL19, IL-17, IL-22, and other cytokines or chemokines, B cells are recruited and accumulate in the tertiary lymphoid structure, and anti-tumor dense aggregates are formed (33, 34). The density of B lymphocytes expressing immunoglobulin (Ig)G, and particularly IgG1, are elevated in tumors and induce antigen-driven reactions with a high affinity. This improves the sensitivity of the humoral immunity of the body, which was shown to relate to positive therapeutic outcomes of TNBC. Vito et al. showed that upregulation of the B cell antigen receptor signaling pathway inhibited the expression of genes encoding nitric oxide synthase 2, arginase 2, IL-1 receptor type 1, and related factors, destroying the inhibitory TME formed by high-density myeloid-derived suppressor cells (MDSCs) in breast cancer, blocking the immune suppression of T cells by MDSCs, and promoting anti-tumor responses of immune cells in the TME (35).

Similar to T cells, regulatory B (Breg) cells are a subset of B cells that suppress immune responses in the TME. They secrete a large amount of IL-10 to negatively regulate T cell immunity. In addition, Breg cells recognize soluble programmed death-ligand 1 (PD-L1), promote B cell differentiation into Breg cells, and stimulate naïve CD4^+^ T cells to differentiate into Treg cell subtypes. A study by Li et al. showed that the percentages of Breg cells and PD-1^+^ Breg cells were the highest among TNBC patients with different breast cancer subtypes, indicating that the prognosis of TNBC was worse than that of the other breast cancer subtypes (36).

#### 2.2.3 NK cells with memory-like functions are expected to become a powerful weapon against anti-tumor immunity

As the first line of defense against tumors, NK cells are roughly divided into two types according to the expression of surface proteins, CD56^high^CD16^dim^ and CD56^dim^CD16^high^. NK cells exhibit two different roles, in which CD56^dim^CD16^high^ NK cells play a cytotoxic role, and release perforin and granzyme B after binding to target cells to mediate apoptosis, while CD56^high^CD16^dim^ NK cells release IFN-γ, tumor necrosis factor, granulocyte-macrophage colony-stimulating factor, and other cytokines to induce other immune cells to attack the target tumor cells. NK cells exert different immune functions as tumor growth progresses. However, long-term exposure to the TME also leads to the inhibition of certain NK cell functions, ultimately promoting the immune escape of tumors (37). Recent studies have found that Socs3^high^CD11b^-^CD27^-^ immature NK cells, which exist only in TNBC, can activate Wnt signaling to achieve tumorigenic effects (38). By targeting PD-L1 with high-affinity NK cells, which is artificially engineered, derived from NK-92, high concentrations of granzyme and perforin granules were released while preserving NK cell receptor expression, which inhibited the growth of MDA-MB-231 cells in TNBC with high expression of PD-L1 (39).

Most studies of tumor immunity focus on adaptive immunity mediated by T or B cells, while only a few studies focus on NK cells in the innate immune system. This is because the turnover rate of NK cells is relatively fast in the human body, and because the memory function of NK cells in the human body remains uncertain. A recent study by Nikzad et al. showed that human liver-resident NK cells displayed long-term antigen-specific memory responses after immunization or viral infection and were not induced by T cells or B cells (40). In addition, the killing ability of memory-like NK cells mediated by IL-12, IL-15, and IL-18 against tumors was significantly enhanced *in vivo* and *in vitro* (41). There are various indications that we are expected to differentiate tumor-associated memory NK cells and maximize their strong killing ability and become a powerful weapon for anti-tumor immunity.

## 3 Adoptive cell therapy brings hope to patients with TNBC

**Figure 2:**
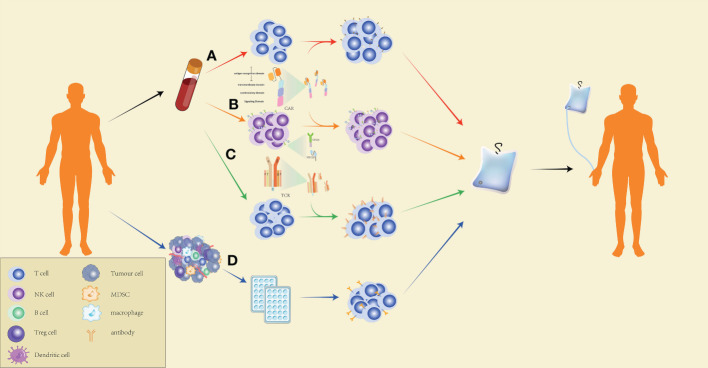
Production process of ACT. **(A)** Preparation of CAR-T cells for CAR-T-cell therapy: peripheral blood mononuclear cells of the patients are extracted by leukapheresis, washed to remove impurities, and specific T cells are isolated using magnetic-bead separation. After activation, viral or non-viral vectors are used to transduce T cells for CAR expression on the cell surface. The CAR-T cells are then expanded to the target dose, and the effectiveness and safety of the cells are checked. CAR-T cells that pass quality control are reinfused into the patients. **(B)** The preparation of CAR-NK cells for ACT is similar to the preparation of CAR-T cells. However, CAR-NK cells exert a more powerful and specific tumor targeting capacity by expressing NKG2D, CD266, and other NK cell surface receptors. **(C)** Preparation of TCR-T cells for ACT: T cells are extracted from the peripheral blood of the patients, followed by delivering specific TCR genes to the T cells through a viral or non-viral delivery system, expanding the TCR-T cells in large quantities, and conducting quality control for the cell culture. The qualified TCR-T cultured cells are reinfused into the patients. **(D)** Preparation of TILs for ACT: Tumor tissues collected from the patients are cut into many small pieces and cultured in a 24-well plate for two to three weeks with medium containing a high concentration of IL-2, followed by selecting TILs which are capable of secreting IFN-γ and culturing them to a therapeutic dose before infusing the cells into patients.

Generally speaking, ACT artificially cultivates lymphocytes that recognize tumors with a high affinity for attacking target tumor cells *in vivo*. Thus, ACT is highly individualized and is divided into four types according to the different mechanisms of action: ACT with chimeric antigen receptor (CAR)-engineered T (CAR-T) cells, ACT with CAR-engineered NK (CAR-NK) cells, ACT with TILs, and ACT with TCR-engineered T (TCR-T) cells (**Figure 2**).

### 3.1 ACT with CAR-T cell technology is the most mature

ACT with CAR-T cells (CAR-T-cell therapy) is an emerging cell therapy. Through genetic engineering, CARs are introduced into the surface of T cells extracted from the patient’s blood. The cells are further cultivated to a certain number and subsequently infused back into the patient’s body, where the modified T cells are now equipped to identify camouflaged tumor cells. CARs are composed of antigen recognition domain, transmembrane domain, costimulatory domain, and signaling domain. After continuous improvement, TRUCKs were raised in the fourth generation of CAR-T-cell therapy. By introducing the nuclear factor of the activated T cell (NFAT)-reactive expression cassette, which can activate cytokines such as IL-12, into CAR-T, and then innate immune cells are attracted to eliminate tumors, bringing hope for solid tumor treatment (42, 43). To 2023, eight CAR-T cell products have been approved for marketing (**Table 1**), indicating that its industrial chain has been approached mature. In addition, CAR-T-cell therapy constantly optimizes production processes and turns its attention to automated production processes that are less costly and less polluting. The successes of CAR-T-cell therapy in clinical aspects also provide new strategies for the production of other ACTs (54, 55).

**Table 1:** FDA- and NMPA-approved CAR-T cell therapies.

Product	Target	Intracellular domain	Sponsor	Agency	Disease	NCT/CTR	Phase	Patients apheresed/treated, n	Response
Tisa-cel	CD19	4-1BB, CD3-ζ	Novartis	FDA	R/R B-ALL	NCT02435849	Phase II	92/75	CR 60%, ORR 81% (44)
					R/R DLBCL	NCT02445248	Phase II	167/115	CR 49%, ORR 53% (45)
					R/R FL	NCT03568461	Phase II	98/97	CR 69.1%, ORR 86.2% (46)
Axi-cel	CD19	CD28, CD3-ζ	Gilead	FDA	R/R DLBCL, tFL, PMBCL, HGBCL	NCT02348216	Phase I/II	111/101	CR 54%, ORR 82% (47)
Brexu-cel	CD19	CD28, CD3-ζ	Gilead	FDA	R/R MCL	NCT02601313	Phase II	74/68	CR 59%, ORR 85% (48)
Liso-cel	CD19	4-1BB, CD3-ζ	Celgene	FDA	R/R DLBCL, FL grade 3, PMBCL, HGBCL	NCT02631044	Phase I	344/256	CR 53%, ORR 73% (49)
Ide-cel	BCMA	4-1BB, CD3-ζ	Celgene	FDA	R/R MM	NCT03361748	Phase II	140/128	CR 33%, ORR 73% (50)
Cilta-cel	BCMA	4-1BB, CD3-ζ	Janssen Research	FDA	R/R MM	NCT03548207	Phase Ib/II	113/97	CR 67%, ORR 97% (51)
Relma-cel	CD19	4-1BB, CD3-ζ	Jw Therapeutics	NMPA	R/R DLBCL	NCT04089215	Phase II	68/59	CR 51.7%, ORR 60.3% (52)
Yescarta	CD19	CD28, CD3-ζ	Fosunkite	NMPA	R/R DLBCL, PMBCL, HGBCL	CTR20181687	Phase I/II	27/24	CR 42%, ORR 79% (53)

*The clinical trials are from clinicaltrials.gov or chinadrugtrials.org.cn.

Tisa-cel, tisagenlecleucel; Axi-cel, axicabtagene ciloleucel; Brexu-cel, brexucabtagene autoleucel; Liso-cel, lisocabtagene maraleucel; Ide-cel , idecabtagene vicleucel; Cilta-cel, ciltacabtegene autoleucel; Relma-cel, relmacabtagene autoleucel; BCMA, B-cell maturation protein; FDA, food and drug administration; NMPA, national medical products administration; R/R B-ALL, relapsed or refractory pediatric B-cell acute lymphoblastic leukemia; R/R DLBCL, relapsed or refractory diffuse large B-cell lymphoma; R/R FL, relapsed or refractory follicular lymphoma; tFL, transformed follicular lymphoma; PMBCL, primary mediastinal B-cell lymphoma; HGBCL, high grade B-cell lymphoma; R/R MCL, relapsed or refractory mantle cell lymphoma; R/R MM, relapsed or refractory multiple myeloma.

However, CAR-T-cell therapy also has disadvantages, such as stimulating an excessive release of immune cytokines after cell infusion and causing the accumulation of a large number of activated macrophages, resulting in serious clinical diseases (56). To reduce the occurrence of such events, numerous improved CAR-T-cells have been established. Due to the high expression of epidermal growth factor receptor (EGFR) in TNBC, Lin et al. designed a CAR-T-cell therapy model targeting EGFR and applied it to TNBC cells. The results showed that the granzyme-perforin-poly adenosine diphosphate (ADP) -ribose polymerase (PARP), factor-associated suicide (Fas) -Fas-associated death domain (FADD) -caspase, and IFN-γ signaling pathways were activated in TNBC cells and had a durable and potent inhibitory effect while ensuring safety (57). Similarly, high expression of mesothelin targets were positively correlated with TNBC progression and differentially expressed in normal and tumor cells. Wang et al. used mesothelin as the potential target of a CAR-T-cell therapy model and combined mesothelin with natural killer group 2, member D (NKG2D) protein to construct a dual-target mesothelin in the VHH-NKG2D CAR-T-cell model, characterized by a high activation level and secretion of many cytokines that killed TNBC cells *in vitro* (58).

There are more complex TMEs in solid tumors, which secrete signaling factors to inhibit the anti-tumor effects of CAR-T cells. Based on this, Stüber et al. blocked TGF-β signaling with SD-208, a TGF-β receptor 1 inhibitor, to effectively prevent the exhaustion of receptor tyrosine kinase-like orphan receptor 1-CAR-T cells in TNBC (59).

### 3.2 CAR-NK-cell therapy has demonstrated a high level of safety

The construction of CAR-NK cells continues the research and development ideas of CAR-T cells. NK cells are mainly derived from peripheral blood, umbilical cord blood, stem cells, and NK92 cell lines. CAR-NK cell models are obtained by using viral or non-viral vectors to transduce CARs into NK cells (60).

Hu (61) used tissue factor (TF) as a target for CAR-NK-cell therapy and developed TF-CAR-NK cells containing a human FVII light-chain recognition domain, CD28 transmembrane domain, 4-1BB co-stimulatory domain, and CD3ζ signaling region. TF-CAR-NK cells directly killed TNBC cells, and this effect was enhanced when combined with second-generation TF-targeted immunoconjugates. Jan et al. used low-dose chemotherapy drugs to downregulate the expression of DNA-methyltransferase 1 and to stimulate the demethylation of the promoter of the transporter associated with antigen processing 1 gene, thus promoting the expression of human leukocyte antigen G (HLA-G) on the surface of tumor cells, facilitating the killing ability of HLA-G-CAR-NK cells and causing extensive tumor ablation (62).

Due to the short survival time of NK cells in the body and the reduced cytotoxicity, with the same therapeutic effect there would be fewer side effects than encountered during CAR-T-cell therapy, such as cytokine release syndrome. Therefore, ACT with CAR-NK cells has shown good safety and therapy responses with great potential for further development (63).

### 3.3 The affinity of TCR-T cells is significantly correlated with the major histocompatibility complex

TCR-T cell technology use lentivirus as a carrier to transduce specific TCRs that are then expressed on the surface of T cells of the patients. Although both ACT with TCR-T cells and ACT with CAR-T cells are referred to as TCR redirection technologies, they have significantly different mechanisms of antigen recognition. ACT with TCR-T cells has an absolute dependence on major histocompatibility complex (MHC). CAR-T cells directly bind to tumor surface antigens, while TCR-T cells must be presented and recognized by the α-β chain heterodimer through MHC. This antigen-recognition property means that TCR-T cells are not limited in the selection of target cell antigens and thus are more suitable for the treatment of solid tumors (64).

Nevertheless, TCR-T cells have a low affinity for antigens than do CAR-T cells (65). Improving the affinity of TCR-T cells to antigens has become an urgent need in anticancer therapy. However, the blind pursuit of a high affinity can also bring many harmful side effects in the anti-tumor treatment process. The affinity between the TCRs and peptide-MHC is low, and the combination of the two fails to induce a strong immune response. To solve these problems, Zhao et al. designed a capture project to prolong the interaction duration between T cells and peptide-MHC and to improve the responsiveness of T cells to ligand signals while ensuring a moderate affinity level (66).

The results of a clinical trial of Kimmtrak-related ACT with TCR-T cells showed that the 1-year overall survival rate of patients who received ACT with TCR-T cells increased to 73%, and the relative risk of death was reduced by 49% compared with patients treated with other drugs (67). As a result, Kimmtrak was approved by the U.S. Food and Drug Administration and became the first approved ACT in the world using TCR-T cells for the treatment of refractory solid tumors. In this context, relevant clinical trials for TNBC, such as ACT with TCR-T cells targeting KK-LC-1 (NCT05483491) and MAGE-A1 combined with atezolizumab (NCT04639245), are in the enrollment stage.

### 3.4 ACT with TILs more accurately identifies targeted tumor cells

ACT with TILs was first demonstrated and applied to the treatment of melanoma by Rosenberg et al., with satisfactory results (68). Unlike CARs, the TILs of this ACT were mainly derived from the tumor tissues of the patients, and TILs capable of secreting IFN-γ in the tissue were extracted and selected for cell culture, which were finally injected into the patients (69). Compared with ACT with CAR-T cells and ACT with TCR-T cells, ACT with TILs has certain advantages and is more suitable for the treatment of solid tumors. T cells that recognize many surface antigens of cancer cells are key for ACT with TILs to attack tumors. In a previous study, 10 out of 13 volunteers who had received other therapeutic regimens but still had metastatic melanoma were infused with TIL products. Among the 10 patients, 50% achieved clinical efficacy, in which 2 patients were in complete remission and 3 patients achieved partial remission. Notably, neoantigen-specific T cell populations, such as RBM12, ENTPD4, VARS, and RAD51AP1-002, emerged and persisted in the peripheral blood of all 10 patients after TIL infusion (70).

The efficacy of ACT with TILs has also been demonstrated in other solid tumors. Standard treatments (e.g., chemotherapy and radiotherapy) are not effective because of the extremely low immunogenicity of metastatic breast cancer (especially TNBC), while ACT with TILs effectively solves this problem. Zacharakis et al. selected 42 patients with metastatic breast cancer who were injected simultaneously with short-term cultured TILs and pembrolizumab. One patient with TNBC had complete regression of liver, lymph node, and mediastinal metastases after 66 months of treatment, and the disease was controlled thereafter by only requiring surgery (71). To date, ACT with TIL therapy for TNBC is now in the stage of recruiting volunteers (NCT04842812 and NCT04111510), fully showing the tremendous potential of this therapy.

## 4 Summary and outlook

As the subtype of breast cancer with the worst prognosis, TNBC has unsatisfactory curative effects after radiotherapy and chemotherapy, and therefore, new treatment options are needed. As the special soldiers in the TME, TILs play an important role in the process of identifying and killing target tumor cells, which has led to the development of immunotherapies. Among those immunotherapies, ACT is a new star and has achieved good results in recent years. The outstanding achievements of CAR-T-cell therapy in hematological tumors and the promising effects of ACT with TCR-T cells in solid tumors have prompted the search for more suitable targets or combination programs for applying ACT to solid tumors. Although issues remain, such as an unknown therapeutic safety, long training period, and expensive cost, with the continuous improvement and development of the technology, ACT that meets expectations will likely be designed in the future, bringing hope to patients with TNBC.

## Author contributions

RL wrote articles and designed diagrams. LC contributed to the revision of the manuscript. Both authors contributed to the article and approved the submitted version.
